# Online Group Psychotherapy: A Systematic Review

**DOI:** 10.1007/s10597-024-01304-4

**Published:** 2024-07-06

**Authors:** Katrina Andrews, Dyann Ross, Tenelle L. Maroney

**Affiliations:** School of Law and Society, University of the Sunshine Coast, Sippy Downs, QLD Australia

**Keywords:** Systematic literature review, Group therapy, Online, Counselling, Psychotherapy

## Abstract

There is a need within counselling and psychotherapy to ensure their ability to respond to endemic presences such as the COVID-19 pandemic, by utilising rapid technological advances without compromising effectiveness. To do so, existing research about online group therapy must be built upon to provide a comprehensive understanding of the efficacious delivery of this therapy modality. Thus, a systematic review was conducted to collate what is currently known in the published and grey literature on online group therapy, using the PRISMA framework. Thirty-three articles were identified as including information regarding facilitating group therapy online. After appraising each article using appropriate quality appraisal tools, a thematic analysis was conducted on the papers, arriving at seven main themes summarising the main findings regarding online versus in-person groups, group planning, counselling micro skills, group processes, ethics, technology, and unique online therapy issues. There is a growing but incomplete body of knowledge that informs the group therapist regarding the ethical delivery of groups online. A more comprehensive review can enable group therapists to feel confident they are across best practice guidelines. Limitations and research implications are considered.

## Introduction

Group counselling and psychotherapy are established therapy modalities involving the purposeful and facilitated sharing of thoughts, emotions and experiences with group members and a group leader. The complex and multiple interactions that occur within the group are understood to enable therapeutic processes for participants (Gullo et al., 2022). These modalities offer numerous benefits to clients, including but not limited to increased accessibility and opportunity for connectedness (Jacobs et al., 2016). Further, recent systematic reviews and meta-analysis conclude that group therapy using cognitive behaviour therapy (CBT), skills development, or mindfulness frameworks are efficacious for people experiencing post-traumatic stress (Schwartze et al., 2019), substance use (Lo Coco et al., 2019), eating conditions (Blood et al., 2020; Moore & Waller, 2023), depression and bipolar disorder (Janis et al., 2021), and anxiety (Barkowski et al., 2020). The coronavirus disease 2019 pandemic (COVID-19) precipitated concomitant social restrictions designed to limit its contagion (e.g. lockdowns, social isolation). A subsequent and significant impact was found on the mental health of the community, with Phiri et al. (Phiri et al., 2021) reporting an increase in mental health burden, higher suicidal ideation, and lower wellbeing in the general public. Also, many group therapists pivoted their group therapy programs to online delivery, using available teleconference technology to do so (e.g., Zoom, Skype) (Weinberg, 2021). This unexpected change in the way group therapy was provided occurred without the research to inform best-practice guidelines for delivering counselling and psychotherapy groups online.

Group therapists attempted to replicate in-person psychotherapy group theory and practice to the online screen environment, but soon found that online group psychotherapy was not the same. Weinberg (2021) provides a summary of the main obstacles to running groups effectively online: the therapist no longer being able to control the group setting to enhance safety and comfort; the loss of cues from body language (other than face); the loss of therapist presence; and the increased chance of distractions, all of which can detract from group effectiveness if not managed.

Best-practice ideas for online group therapy include: the need for the therapist to spend more time checking that group members attend group therapy in a safe, secure setting that is free from distraction; to pay more attention to facial expressions in order to read the (online) room; the use of therapist self-disclosure to increase online presence; and paying more attention to the meaning of various distractions (Weinberg, 2021). This represents a helpful start to the body of evidence needed to ensure the counselling and psychotherapy profession responds to the call to effectively respond to the practice implications of strictures arising from the effects of COVID-19 and its variants (Skegg et al., 2021). The adaptation to online forums was indicative of the importance of being able to respond at the community level (Resnick & Fins, 2021), whilst remaining at the forefront of rapid technological advances without compromising the effectiveness of group therapy (Hanley, 2021).

A systematic literature review was conducted with the aim of completing a comprehensive and robust summary of what is currently known about evidence-based online group therapy in the published and grey literature. It is hoped that the results will highlight what is yet to be known in regard to facilitating therapy and counselling groups ethically and efficaciously in the online space.

## Method

A thematic analysis style of knowledge synthesis was adopted (Kastner et al., 2012) to ensure the findings reflect current, prominent, and recurring themes captured from mixed literature (methodologies, study designs, published, and results of quality appraisals). Thematic analysis involves the identification of key themes and ideas across a body of literature and summarising the main findings under thematic headings, which inform the key points of relevance (Kastner et al., 2012).

### Search Strategy

The search strategy adopted was the PRISMA-S checklist (Rethlefsen et al., 2021). To access published literature, the following databases were accessed: Google Scholar, PsycNET, Psychiatry Online, ScienceDirect, ProQuest, Web of Science, and Scopus. To access grey literature (i.e., unpublished literature), the following databases were accessed: Google, OpenGrey (https://opengrey.eu/), Base (https://www.base-search.net/), and MedNar (https://mednar.com/mednar/desktop/en/search.html). Finally, the websites of the professional associations related to group therapy were reviewed for relevant literature (Australian Association of Group Psychotherapists, AAGP; International Association of Group Psychotherapy, IAGP; & American Group Psychotherapy Association, AGPA).

**Fig. 1:**
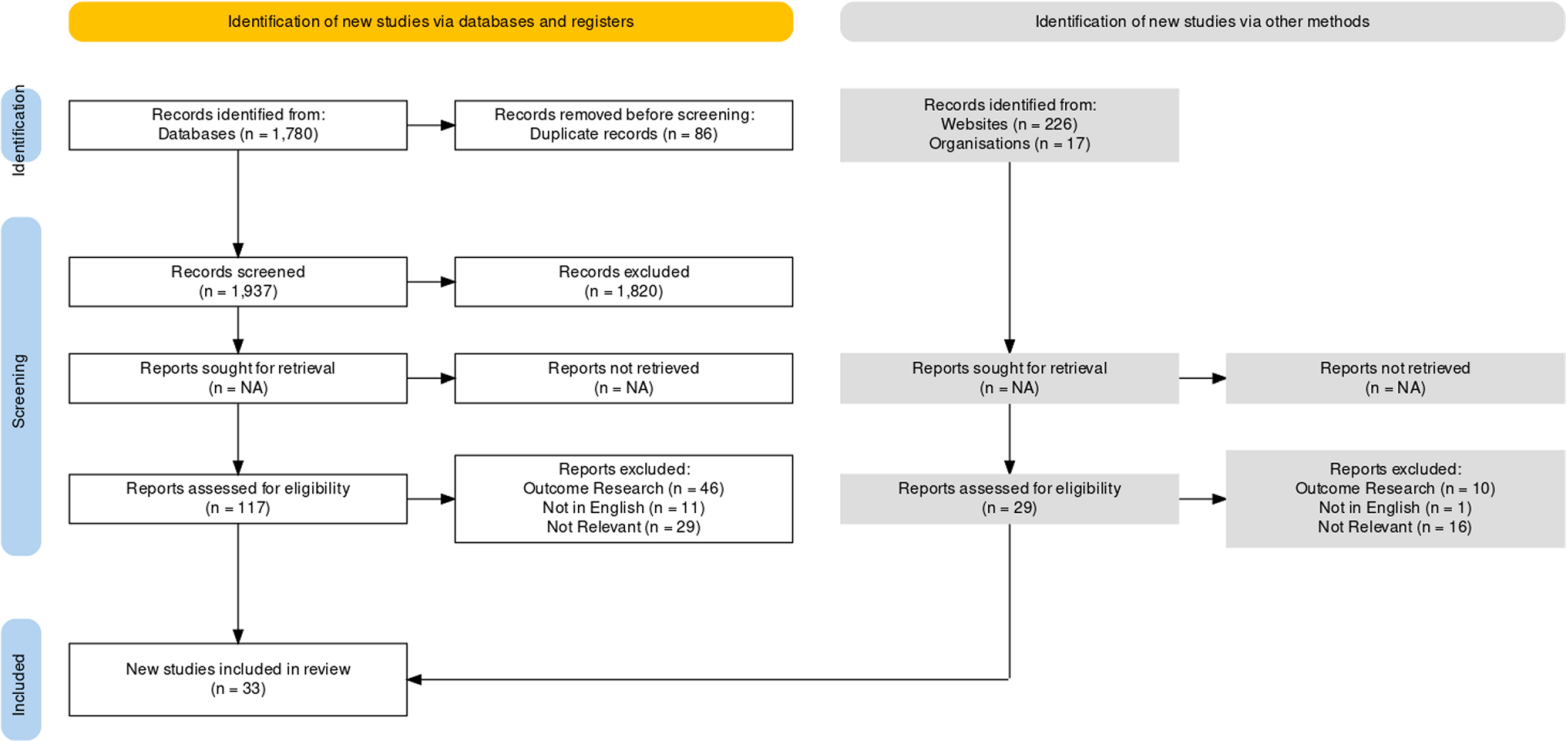
Prisma flowchart detailing selection of included studies. Adapted from Haddaway et al. (2022)

The following search terms were used in the above databases: online, group, therapy, psychotherapy, counselling (British and American spelling). Inclusion criteria included: English, published between 2012 and 2022. Exclusion criteria included: support groups, education/teaching groups. For the purpose of this paper, mental health professionals comprised qualified counsellors and therapists within the disciplines of counselling, psychology and social work. This resulted in an initial 1780 papers from the published literature databases, and 243 papers from the grey literature databases (Fig. 1).

The initial 2024 papers were next screened for duplication, resulting in the removal of 86 records. The resultant 1937 records were then screened for relevancy via title, resulting in the exclusion of 1820 published papers and 214 grey literature papers. The surviving 117 published papers and 29 grey literature papers were next screened via abstract. This resulted in the further exclusion of 56 papers because they summarised the findings from outcome research, 12 papers because they were not published in English, and 45 papers as they were deemed not relevant. This resulted in a final list of 33 papers (see Table 2) that were included in the quality appraisal stage of the current knowledge synthesis.

### Quality Appraisal

Due to the inclusion of mixed studies (by design, methodology, published, grey, and non-peer reviewed), a number of quality appraisal tools needed to be adopted to ensure the appraisal of each paper was appropriate for its type (Refer Table 1).

**Table 1:** Quality appraisal tools

**Tool**	**Type of publication**
JBI Checklist of Quasi-Experimental Studies (Non-Randomised Experimental studies) (JBI, 2023)	Quantitative quasi-experimental (between- or within- group) research design studies
JBI Checklist for Analytical Cross-Sectional Studies (JBI, 2023)	Quantitative correlational research design studies
JBI Checklist for Qualitative Research	Qualitative studies
JBI Checklist for Text and Opinion (JBI, 2023)	Book chapters and opinion papers
Mixed Methods Appraisal Tool (MMAT; Hong et al., 2018)	Mixed method studies
The AACODS Checklist (Tyndall, 2010)	Grey literature

Each paper was appraised independently by two of the authors. After appraising each paper using the most appropriate appraisal tool, each author rated the paper as either strong (+++), satisfactory (++), weak (+), or exclude (ex). When completed, the authors compared the results and any discrepancies in ratings were identified. These identified papers were then appraised by the third author, and the final rating was adopted. Refer Appendix A: Table 2 for the results of the quality appraisal process.

### Data Analysis

The surviving 33 papers were then analysed using thematic analysis for systematic reviews (Mays et al., 2005), following the six-step reflexive thematic analysis (RTA) process espoused by Byrne (Byrne, 2022). All papers were analysed in their entirety except for empirical articles, in which the results section only was analysed. NVivo 20 (2022/23 license) was adopted to assist in the coding process.

### Reflexive Practice

Reflexive practice serves to acknowledge the positionality of the researcher as an active agent in the analytical process (Trainor & Bundon, 2021). The power relationships in research are less evident when secondary sources are involved, but we still worked to ensure there was no bias in our data analysis to be fair to all researchers’ work. As suggested by Byrne (2022), the authors engaged in reflexive practice in two ways. Firstly, each author kept a journal of their own thoughts, reactions, expectations, and ponderings during the data analysis stage of the study. This ensured each author engaged in a hermeneutic process whilst analysing the data, ensuring they arrived at codes, categories and themes that describe the phenomenon objectively as much as possible, and without personal bias or expectations (Smythe & Spence, 2012). Secondly, each author independently analysed the data, and then collaborated with each other regarding their own results, looking for similarities and discrepancies in the list of final themes. Discrepancies in themes were considered in three ways: (a) by considering whether the discrepant theme was captured by the second author using another term, (b) by considering whether the discrepant theme was captured by the second author as a subtheme, or (c) by considering whether the discrepant theme captures distinctive and valuable ideas not otherwise captured in existing themes. Using these “rules”, the final list of themes was arrived at.

## Results

Seven major themes were arrived at and are summarised in Appendix A: Table 3. The table displays each of the major themes and the sub-themes contributing to each theme. Text selections from the primary source are offered to illustrate the theme being defined (in italics), and a coding system is offered to locate the text selection within the primary source (e.g., the primary source as listed in the reference list, and the page number in the primary source). Finally, emphasis in each theme is given to those papers rated strongest during quality appraisals.

### Theme 1: Online vs F2F

Theme 1 captured overall opinion found in the literature regarding the credibility and acceptability of online group therapy being a viable therapy modality from both the group member and group facilitator perspective. When compared to F2F group therapy, online group therapy was unanimously experienced as very different. However, some papers reported that online group therapy is inferior to in-person group therapy. An in-depth summary is presented in the following subthemes.

#### Subtheme 1.1: Group Member Viability

This subtheme captured codes addressing member acceptance and benefits of online group therapy. Overall, many authors reported that online group therapy is considered beneficial by group members for many reasons including that many members reported feeling less judged online (Hamadi et al., 2022), and the online group atmosphere remained positive (Adamlje & Jendricko, 2020; Arrow et al., 2021; Datlen & Pandolfi, 2020). Online group therapy was also reported as more convenient and accessible (Ellet et al., 2022; Kozlowski & Holmes, 2017; Weinberg, 2020), with less stigma (Weinberg, 2020), and more options for participation (Datlen & Pandolfi, 2020; Shaw, 2020). A quote from Kozlowski and Holmes (Kozlowski & Holmes, 2017) demonstrates this when they state that online group therapy *creates a sense of universality that eliminates geographical boundaries, and perhaps gender and culture* (p. 5).

However, authors equally stated that members reported feeling a lack of group presence and connection (Kozlowski & Holmes, 2014) when attending group therapy online and described the online group therapy experience as anxiety provoking (Adamlje & Jendricko, 2020), frustrating (Armington et al., 2020), and artificial (Kozlowski & Holmes, 2014). For example, Kozlowski and Holmes (Kozlowski & Holmes, 2014) stated that members felt *they did not really feel like they were present in a counselling group as compared to a face-to-face group experience* (p. 289). Finally, authors noted that online group therapy is not suitable for certain populations, including older persons and those suffering complex trauma (Weinberg, 2020).

#### Subtheme 1.2: Group Leader Viability

As with members, online group therapists were also mixed in their acceptance and benefits of this modality. Regarding group therapist acceptance, authors generally stated that online group therapy offered advantages to group therapists including better observation of member facial expressions to infer affect and intrapsychic reactions (Adamlje & Jendricko, 2020), and the flexibility of different ways of communicating, including the chat function and the whiteboard (Hung et al., 2021). Furthermore, authors commented that online group therapy has a greater sense of universality compared to in-person therapy groups (Weinberg, 2019a) and is a preferred medium for therapy for some populations including those with dismissive-avoidant attachment styles or social anxiety (Adamlje & Jendricko, 2020; Hamadi et al., 2022; Pipkin et al., 2022; Weinberg, 2020). Authors declared that online group therapists perceive themselves as more present and empathic compared to facilitating groups in-person (Gullo et al., 2022). Finally, it was reported that group completion rates are improved online (Dijk et al., 2020; Ellet et al., 2022; Gürcan & Yildiz, 2021; Hamadi et al., 2022; Joshi et al., 2021), with reasons for absenteeism commonly being unavoidable personal problems or internet connectivity (Adamlje & Jendricko, 2020; Amulya & Manickam, 2020). These group therapist reported benefits are represented well by Adamlje and Jendricko (2020), when they stated that online group therapists are able to *see all the group members simultaneously and up-close, giving them the opportunity to study their facial expressions and use them to form conclusions on how other members felt, which they would often comment upon* (p. 298–299).

Despite the above reported benefits for group therapists, authors also reported on some drawbacks, including that online group therapy is more demanding in that it requires more focus (Adamlje & Jendricko, 2020; Weinberg, 2021) which can be fatiguing (Lovko et al., 2021) and online group therapists feeling less competent in their role (Gullo et al., 2022; Lovko et al., 2021; Shaw, 2020). Further, authors reported that the online format may hinder traditional group processes (Adamlje & Jendricko, 2020; Kozlowski & Holmes, 2014; Weinberg, 2020), particularly non-verbal communication (Weinberg, 2020). A quote from Gullo et al. (2022) summarises these disadvantages well, when she stated that there are *challenges of working through conflict, managing avoidance, and observing non-verbal communication […] feeling nervous looking at one’s own image, as well as therapists feeling nervous and discomfort during the online group session* (p. 110).

### Theme 2: Group Planning

Theme 2 captured what is considered important logistically to optimise the online group therapy experience. Kozlowski and Holmes (2017) emphasised this when they stated that group leaders *should consider the uniqueness of the online environment as opposed to trying to simply recreate face-to-face group counselling* (p. 17).

#### Subtheme 2.1: Scheduling

Online group therapy sessions often follow the same frequency and length as in-person group therapy sessions (Adamlje & Jendricko, 2020; Pipkin et al., 2022). However, some studies hint that online group sessions might also require additional time for member introductions (Kozlowski & Holmes, 2017), informed consent (Whittingham & Martin, 2020), and thus session duration should be extended (Datlen & Pandolfi, 2020). Further, more time should be expected to achieve active member participation and group cohesion (Hung et al., 2021; Weinberg, 2019b).

#### Subtheme 2.2: Screening

Several authors emphasised the need first for an online survey comprising demographic details and group expectations followed by pre-group interviews (Amulya & Manickam, 2020; Joshi et al., 2021; Maheu, 2020; Whittingham & Martin, 2020), similar to screening procedures for in-person group programs. Further, Adamlje and Jenko (2020), Hamadi et al. (2022), and Pipkin et al. (2022) emphasised group size ranging from 8 to 12 participants, to up to 17 members (Gürcan & Yildiz, 2021).

#### Subtheme 2.3: Pre-Group Preparation

Van Dijk et al. (2020), Maheu (2020), Parks (2020), and Whittingham and Martin (2020) advised that pre-group meetings and/or screening should be conducted to determine participant suitability, set group expectations, goals and guidelines prior to commencing therapy. Weinberg (2019a, 2021), Maheu (2020) and Shaw (2020) added that these pre-group meetings should also be used to familiarise members with the adopted technology including common technical issues and their solutions, internet etiquette (Weinberg, 2019a, 2021), how to set up their physical space (Maheu, 2020), and any therapy supplies (in the case of art therapy) (Shaw, 2020). Parks (2020) shared a quote regarding this, stating the pre-group meetings was important to ensure there was *congruence in [participants’] beliefs that these technologies are at least appropriate for the task at hand* (p. 120).

#### Subtheme 2.4: Online Group Leadership

To navigate the added responsibilities imposed by leading groups online, Joshi et al. (2021), Shaw (2020), and Weinberg (2020) recommended that online groups should be co-facilitated, with the co-facilitator moderating the content shared by members via the alternative communication methods, such as the chat function. Weinberg (2020) added that co-facilitators should not communicate to each other via text messages due to it adding a distraction to the group and its non-transparency. Instead, Weinberg (2020) and Shaw (2020) recommended co-facilitators *verbally communicating to each other in the presence of the group members* (Weinberg 2020, p. 207).

#### Subtheme 2.5: Online Group Rules

Adamlje and Jenko (2020) and Weinberg (2019a) stressed that the online group rules are identical to those expected in in-person groups, but the emphasis on the maintenance of these group rules is greater in the online setting. Authors noted added group rules, unique to the online context including: showing appreciation to each other non-verbally using the virtual reaction function (Hung et al., 2021); not having out-of-group relationships, even online (Weinberg, 2019a); no recording of sessions (Dijk et al., 2020; Maheu, 2020); adhering to group-established policies for instances such as disconnection and confidentiality breaches (Whittingham & Martin, 2020); ensuring cameras and microphones remain on to stimulate communication between group members (Hung et al., 2021; Weinberg, 2019b); the use of the whiteboard function (in Zoom or Google Meets) so members can express ideas through writing and drawing (Hung et al., 2021); talking in some type of order to ensure members do not interrupt each other (Gürcan & Yildiz, 2021); joining from a quiet room with full privacy and no interruptions, including phone calls, emails, or texting (Weinberg, 2019b); and joining from the same physical location each session (Weinberg, 2019b).

### Theme 3: Group Processes

This theme captured the material found in the literature that comments on how known and accepted group processes translated in the online context. Four subthemes emerged in this theme, as discussed below.

#### Subtheme 3.1: Therapeutic Presence

Geller et al. (2022) defined therapeutic presence as a *way of being with a client that optimises the doing and technique of therapy […] being fully in the moment on a multitude of levels including physical, emotional, cognitive, relational, and spiritual* (p. 2). Presence helps the client feel felt, met, and understood, and is the necessary precursor to a healthy therapeutic alliance, and thus effectiveness (Geller et al. 2022, p. 2). Amulya (2020) concluded that a therapeutic presence is possible with online group therapy, but the quality of therapeutic presence online is diluted (Trachtenberg et al., 2020; Weinberg, 2019a, 2020, 2021).

Weinberg () offered ways that a group therapist can increase therapeutic presence online, including: (a) appropriate self-disclosure, (b) acknowledging mistakes and empathic failures, and (c) utilising imaginative exercises. For example, Weinberg (2019c) stated that self-disclosure can *overcome the ‘screen barrier’ and helps the group members feel that they can also stay sensitive and compassionate despite the limitations of online connection* (p. 184).

#### Subtheme 3.2: Group Cohesion

Group cohesion is understood as the group’s connectedness, another critical factor in the group’s effectiveness (Forsyth, 2021). There was consensus in the literature that, despite a group being online, members still report a sense of connectedness (Amulya & Manickam, 2020; Datlen & Pandolfi, 2020; Ellet et al., 2022; Hamadi et al., 2022), and alliance (Amulya & Manickam, 2020; Weinberg, 2020) which strengthened as the group progressed (Joshi et al., 2021). Gullo et al. (2022) stated that therapists found it easier to foster group cohesion online, and Weinberg (2021) added its just takes longer to develop. Trachtenberg et al. (2020) summarised that group cohesion can be maximised by pre-screening participants for appropriateness, encouraging non-linear communications between group members, as well as encouraging imagination, and Armington et al. (2020) reported that members perceived tenderness and warmth from other members when they reached out toward the screen.

Hung et al. (2021) cautioned that group cohesion can also be easily lost if an online therapist misunderstood messages or facial expressions, or if members have increased anxiety and frustrations leading to negative transference within the group (Adamlje & Jendricko, 2020). However, Adamlje and Jenko (2020) added that should the therapist be able to repair these empathic tears online, the result is *a greater intimacy between members, increased faith in the group, and deeper sincerity* (p. 323).

An exemplary excerpt of a therapist using imagination to promote online group cohesion was offered by Trachtenberg et al. (2020) when he shared: *I am imagining us joining around our big old coffee table. Everyone shaking off coats, and getting warm drinks! Welcome everyone!* (p. 316).

#### Subtheme 3.3: Holding Environment

In regard to creating a safe holding environment in the online context, Weinberg (2020) stated that the therapist loses control of this in the online context, since members join from their own homes. However, the therapist can assist members in ensuring a safe holding environment by educating the group member of the importance of joining from a quiet and confidential space with minimal to no distractions (Weinberg, 2020) and having technical expertise (Weinberg, 2019b), delivered in a timely manner (Weinberg, 2019c). To demonstrate this, Weinberg (2019c) stated that *the group therapist should compensate for the loss of the control on the setting by developing suitable online administrative functions* [p. 178].

To maintain the holding environment, Weinberg and Rolnick (2019) and Weinberg () stressed that online therapists need to *explore and not ignore* even the smallest of incidents that occur during sessions (Weinberg & Rolnick, 2019, p. 171). They explain that the simple act of a client’s cat appearing on the screen, or technical glitches, can have several meanings for example, and this needs to be called out and explored.

#### Subtheme 3.4: Member Dynamics

Adamlje and Jenko (2020) detailed that member dynamics in online groups can be similar to in-person groups but with some added novel member dynamics as well, including increasingly longer and more frequent periods of silence generally occurring around the 9th or 10th online session, blaming obstacles to communication on the technology as a way to avoid discussing sensitive material (Weinberg, 2021), increased direct and indirect anger towards the group therapist (Adamlje & Jendricko, 2020), and members finding it easier to be inauthentic within the online space (Kozlowski & Holmes, 2014). Conversely, due to the lack of informal contact between members before and after groups, online member dynamics is increasingly restrained resulting in a more deeply immersed experience benefiting the group and its growth (Adamlje & Jendricko, 2020; Hung et al., 2021).

The process of drawing-out quiet members is a group process of more importance in the online group format. According to Kozlowski and Holmes (2017), *group leaders should be assertive in asking clients how they feel and their reactions to what is being shared, so as to engage members more completely* (p. 19).

### Theme 4: Micro-Skills

This theme captured utterances in the literature that outlines how counselling microskills are altered due to the online context. Two subthemes were identified in this category.

#### Subtheme 4.1: Verbal Communication

Kozlowski and Holmes () and Adamlje and Jenko (2020) both identified that online communication styles differed compared to face-to-face communication styles, describing it as linear (Kozlowski & Holmes, 2014), meaning that members waited for their turn to talk, which either resulted in members feeling cut off, or members appreciating the structure this communication style created (Kozlowski & Holmes, 2014). This question-and-answer pattern of communication was also observed by Gürcan and Yildiz (2021), Hung et al., (2021), Datlen and Pandolfi (2020), and Weinberg (2019b).

Adamlje and Jenko (2020) further reported that over time, members soon learned to adjust how they communicated online to optimise communication, including adding more frequent pauses to account for time lag, and normalising the awkward silence (lag) between utterances (Kozlowski & Holmes, 2017). As a result of this observation, Weinberg (2019b) cautioned that online group leaders should avoid the*’star-shaped’ communication structure (when leader is in centre of communication), and to encourage members to respond to each other, without prompting from the leader* (p. 207).

#### Subtheme 4.2: Non-Verbal Communication

It was unanimous in the literature that the lack of ability to observe gestures and body language, or make eye contact (Weinberg, 2019c), resulted in some communication being missed, reduced, or misinterpreted (Adamlje & Jendricko, 2020; Gullo et al., 2022; Hung et al., 2021; Kozlowski & Holmes, 2014; Parks, 2020). Despite this, Kozlowski and Holmes (2014) observed that members soon adjusted to the limited non-verbal communication, by *focusing on communicating content and [using] tone to infer feelings and reactions* (Parks, 2020, p. 119).

Other ways that authors reported to overcome the inability to infer from non-verbal communication included psychoeducation about the challenges to accurately detect emotions in the online environment (Parks, 2020), encouraging clients to verbally report their body sensations (Kozlowski & Holmes, 2017; Weinberg, 2020), or move (closer or further way) from the screen (Weinberg, 2020). Kozlowski and Holmes (2017) added that members should be encouraged to express themselves verbally and to speak out when they are feeling disconnected, and group therapists should feel compelled to address any missing or confusing information through narrative discussion, allowing for genuine dialogue regarding the clients’ feelings and experiences. Kozlowski and Holmes (2017) offer a statement, demonstrating the therapist verbalising what is usually non-verbal, as follows:

*Leaders need to intentionally alert members when they are applying silence in the group*. *For example, leaders deliberately ask a member or members to take time to process their thoughts and feelings about an issue after it is presented in the group. This separates therapeutic silence from inadvertent lag time* (Kozlowski and Holmes 2017, p. 18).

### Theme 5: Ethics

Three subthemes regarding ethical considerations specific to online group therapy were identified, as summarised below.

#### Subtheme 5.1: Safety

Both Adamlje and Jenko (2020) and Hamadi et al. (2022) discussed incidences of members cancelling their participation due to scepticism, suspiciousness, discomfort, and anxiety. This reduced sense of safety by online group members was also reported by Kozlowski and Holmes (2014) and Holmes et al. (2015).

Joshi et al. (2021) and Armington et al. (2020) observed that members would adapt to their discomfort online by discussing sensitive topics via the chat function as it offered a degree of anonymity. Further, Armington et al. (2020) recounted that the discussion of anxious thoughts by each member during the first meeting had the effect of lowering their anxiety. Finally, Shaw (2020) recommended including a protocol that members follow should they become distressed, such as being *called on the phone by one of the facilitators and digitally sent out of the room for a few minutes* (p. 213).

#### Subtheme 5.2: Boundary Breakdowns

Adamlje and Jenko (2020), Weinberg (2021), and Kozlowski and Holmes (2014) discussed that online group members were more casual, relaxed and distracted compared to members of in-person groups. This observation was also extended to the members appearance, with Weinberg (2021) stating that members *would be unshaven/unwashed, wear pyjamas, or wear inappropriately revealing or sexualised apparel* (p. 36).

Adamlje and Jenko (2020), Weinberg (2019c, 2020), and Whittingham and Martin (2020) stated that these issues need to be addressed, since they would not be tolerated in an in-person group. Weinberg (2019c) recommended a whole-of-group discussion about the noticed boundary breakdown, so the group therapist does not carry this burden on their own.

#### Subtheme 5.3: Confidentiality

It was unanimous in the majority of the found literature that ensuring confidentiality in the online group space is more difficult (Hung et al., 2021; Maheu, 2020; Weinberg, 2019c, 2021; Whittingham & Martin, 2020). Besides the risk of being overheard by persons residing in the same physical location, Kozlowski and Holmes (2014) and Maheu (2020) added that other breaches to confidentiality unique to the online group experience includes members taking screen shots or record[ing] sessions, and post[ing] these to social media sites (Kozlowski and Holmes 2014, p. 289], or using recorded material to *share the identity of or blackmail the group or a specific member* (Whittingham & Martin, 2020, p. 2).

To address these added confidentiality concerns, subtheme 3.3 above summarised what online group therapists can communicate to group members to prepare a safe holding environment. Added to this, Whittingham and Martin (2020) discussed letting members *wear a disguise or block their faces using non-threatening, preapproved masks that still allows for speech to be heard […] using a fictitious name or only an initial as on-screen identification* (p. 3).

### Theme 6: Technology

Not surprisingly, technology and its use were a common theme in much of the found literature. Theme 6 captured four subthemes, as summarised below.

#### Subtheme 6.1: Software

Seventeen papers reported on the platform adopted to facilitate the online group therapy programs, with the majority of these papers reporting Zoom as the preferred platform (Adamlje & Jendricko, 2020; Amulya & Manickam, 2020; Armington et al., 2020; Belem, 2021; Ellet et al., 2022; Gürcan & Yildiz, 2021; Joshi et al., 2021; Weinberg, 2019a). Reasons Zoom was preferred include the access to gallery view, screen share options, waiting room, convenient, and accessible (Amulya & Manickam, 2020; Weinberg, 2019b), and HIPAA-compliant (Joshi et al., 2021; Whittingham & Martin, 2020). Other platforms include Microsoft Teams (Hamadi et al., 2022; Pipkin et al., 2022; Shaw, 2020), WhatsApp (Datlen & Pandolfi, 2020) and Webex (Dijk et al., 2020).

#### Subtheme 6.2: Hardware

Adamlje and Jenko (2020) and Whittingham and Martin (2020) both reported a minimum that a member needs to be able to be successful when attending online group therapy, including a good internet connection, good quality devices, headphones, and a microphone. Weinberg (2019b) recommended members to attend online groups using a tablet or laptop, but not on mobile phones given the limited gallery view function.

#### Subtheme 6.3: Technological Issues

Common technological issues faced by group leaders included much time dedicated to resolving technological issues (Adamlje & Jendricko, 2020), unclear video and/or audio (Amulya & Manickam, 2020), disrupted internet connectivity (Amulya & Manickam, 2020; Joshi et al., 2021), and technology glitches (Ellet et al., 2022). Effects of these issues on the group include a disturbed session flow and member discomfort (Amulya & Manickam, 2020), impatience (Hamadi et al., 2022), and paranoia (Ellet et al., 2022). Solutions to these inevitable technological issues included discussing the meanings attached to these disruptions, including the emotions that these disruptions evoke (Amulya & Manickam, 2020; Weinberg, 2021), including a present-but-absent scenario requiring the leader to call out to them to check their presence from time to time (Amulya & Manickam, 2020), connecting to the group five minutes before start to address any technical issues (Adamlje & Jendricko, 2020), using the first 30 min of a session to address any technological issues (Hamadi et al., 2022), allowing the private question feature so members can communicate any technical difficulties (Maheu, 2020), and having a standard protocol for members to follow should the connection be lost (Maheu, 2020).

#### Subtheme 6.4: Chat Function

Weinberg (2019b) and Maheu (2020) both stressed that online group facilitators limit or disable the chat function during a group meeting, as it distracts members from the group process and can become an informal communication network that is hidden from the group facilitator. If enabled, it should only be used to overcome technical difficulties, and Datlen and Pandolfi (2020) encouraged the use of emoji’s or stickers as this enables members to express emotions more easily.

### Theme 7: Unique to Online

Theme 7 summarised what has been captured in the literature that is unique to facilitating online counselling/therapy groups. Four subthemes were identified.

#### Subtheme 7.1: Role Confusion

Kozlowski and Holmes (2014, 2017) reported that it was common for online group members to adopt the role of student and wait to be called on to talk and share. Similarly, Kozlowski and Holmes (2014, 2017) reported that it was common for online group leaders to adopt the role of teacher, working hard to get members to participate. Kozlowski and Holmes (2017) added that online group leaders should encourage crosstalk among members to overcome role confusion.

#### Subtheme 7.2: Embodied Presence

Weinberg (2021) and Trachtenberg et al. (2020) discussed that the experience of embodiment in online groups as not being absent, but rather different. Specifically, our own bodily sensations remain intact [during group], but our interactions between bodies is lacking (Weinberg, 2021). Weinberg (2021) explained that in online group therapy, the treatment is a *nonbody treatment* (p. 85) since the physical bodies of members and the leader are only perceived visually and not through other senses, and not even the full body can be seen, but only the faces of members and the leader. Due to the lack of physical presence in online groups, Armington et al. (2020) noted that online group members were *reaching out [toward the screen] with their hands* resulting in group members perceiving tenderness and warmth [from other members] and an increased connection (p. 2).

#### Subtheme 7.3: Members’ Physical Space

Adamlje and Jenko (2020) stated that members mostly attended the online group from their own homes, with occasional other locations being work, or on vacation. When in their own home, members are encouraged to ensure the location is private (Pipkin et al., 2022; Whittingham & Martin, 2020), and is a space dedicated to therapy (not a living or bedroom) (Dijk et al., 2020), since other group members may feel uncomfortable witnessing a member's private space (Pipkin et al., 2022).

Kozlowski and Holmes (2017), Pipkin et al. (2022), van Dijk et al. (2020) and Whittingham and Martin (2020) all stated that the leader needs to be active in educating members of the importance of a private and dedicated therapy space in their home, with a ‘do not disturb’ sign on the closed door (Maheu, 2020). If a distraction occurs, Weinberg (2019c) stated that the distraction needs to be part of the group process and discussed.

Weinberg (2019c) added that leaders should be aware of the decoration [in the background] and explore its meaning when appropriate, since members may deliberately manipulate their background (Kozlowski & Holmes, 2014) to *portray their individuality in a controlled, simulated way, [which added] to the artificial feel of the group experience* (Kozlowski & Holmes, 2014, p. 288).

#### Subtheme 7.4: Self/Others on Screen

Multiple authors discussed how members find their own image on the screen distracting and even dysphoric, resulting in either reduced group participation, or altered group communication (Kozlowski & Holmes, 2014, 2017; Pipkin et al., 2022; Shaw, 2020; Vaimberg & Vaimberg, 2019; Weinberg, 2021). Weinberg (2019b) recommended members turn off their view of themselves on the screen. Alternatively, Adamlje and Jenko (2020) reported that group members seeing each other simultaneously and up-close provided opportunity to study facial expressions, and use these expressions to form conclusions on how others felt, which they could then offer a comment about.

### Subtheme 7.5: Self Care

Attending online therapy groups also have the added risk of zoom fatigue (Amulya & Manickam, 2020). To minimise zoom fatigue, Amulya (2020) recommended adding sufficient breaks, and Armington et al. (2020) recommended reminding group members to engage in self-care activities after group.

## Discussion

Worldwide events such as the COVID-19 pandemic necessitated mental health professionals, including group therapists, to rapidly pivot their talk therapy to an online screen environment (Weinberg, 2021). However, there was little to no research evidencing the best-practice guidelines for delivering psychotherapy groups online. According to Weinberg (2021) group therapists discovered many obstacles to success, including decreased ability to create a safe space, loss of body language cues, loss of therapist presence, and increased distractions. He offered some suggestions for ethical online group therapy, including therapists spending more time ensuring members join the online group from a safe and secure setting that is free from distraction; therapists paying more attention to facial expressions in order to read the (online) room; therapists using self-disclosure to increase online presence; and therapists paying more attention to the meaning of various distractions. However, the influence of contextual factors on the efficacy of providing group therapy remains a potential challenge. It is widely accepted that the future is largely uncontrollable and unpredictable (OECD (Organisation for Economic Co-operation and Development), 2019). In this regard, the flexibility to change modes of offering group therapy needs to be supported with best-practice research (Skegg et al., 2021).

Thus, the current systematic review was completed in order to arrive at a comprehensive and robust summary of what is currently known about facilitating online group therapy. It is believed that this knowledge can enable our current group therapists to continue their necessary work regardless of unknown future disruptive global events preventing in-person group therapy attendance. This will enable mental health professionals to respond to a recent call by Dishon and Gilead (2021) to be adaptable in their skillset.

The seven themes identified in the systematic literature review demonstrate that there is indeed a body of knowledge that can be accessed to inform the ethical facilitation of group therapy in the online context, above and beyond what was initially summarised by Weinberg (2021). This includes ensuring members attend the online group therapy in a safe and secure setting which is free from distractions, paying added attention to facial expressions on the screen, using therapist self-disclosure to develop the therapist presence online, and paying attention to the meaning given to the various distractions during the group session. The current systematic review has been successful in collating robust knowledge and skills pertaining to planning the online group program (scheduling, screening, pre-group preparation, group leadership models, and added group rules), and the required software and hardware for successful online groups.

Further, the systematic literature review has elucidated on added ethical issues to consider in online group therapy (besides safety). In particular this included the adopted term boundary breakdowns, referring to the relaxation of appearance and presentation by online members compared to in-person groups (Adamlje & Jendricko, 2020; Kozlowski & Holmes, 2014). Added to this, the current review uncovered some added risks to confidentiality that need to be managed (Adamlje & Jendricko, 2020; Whittingham & Martin, 2020).

Not surprisingly, the systematic review uncovered how common in-person group processes and practices do not translate well in online group therapy, and how they have been adjusted to suit the online context, including group processes and therapeutic micro skills. Additional to the increased use of therapist self-disclosure to increase therapist presence in the online space as offered by Weinberg (2021), Weinberg (2019b, 2019c, 2020, 2021) provides further ideas including therapists’ humility for mistakes and empathic failures and the greater use of imagination. Interestingly, some authors stated that group cohesion is easier to establish in the online context (Gullo et al., 2022), although it takes longer to do so (Weinberg, 2021) and is more easily ruptured in the online context (Hung et al., 2021). Trachtenberg et al. (2020) offered suggestions to strengthen group cohesion online, including non-linear communication styles between members, the use of imagination in groups, and members reaching out to the screen with their hands (Armington et al., 2020). The therapists own technical expertise emerged as the online equivalent of creating a safe holding environment for group members (Weinberg, 2019b). Finally, added group member dynamics in the online space included longer and more frequent periods of silence, blaming technology as a way to avoid the discussion of sensitive topics (Weinberg, 2021), increased anger towards the group leader, and deeper dives into topics of discussion compared to in-person groups (Adamlje & Jendricko, 2020).

Therapeutic microskills in the online group context included the linear or star-shaped style of communication between members and leaders (i.e., taking turns and waiting to be called upon) which was experienced by members as unnatural and awkward (Kozlowski & Holmes, 2014, 2017), but also simultaneously appreciated by members for the structure (Kozlowski & Holmes, 2014). Adamlje and Jenko (2020) added that this communication style soon disappears when members adjust to the online context over time. The lack of non-verbal cues was a common theme in the literature, but authors recognised that this was soon compensated for via the use of vocal tone or members verbalising their non-verbal cues (Parks, 2020). Another adjustment was for members to move away from, or closer, to the screen (Weinberg, 2020).

Of most interest was the theme summarising issues that are unique to online group therapy, which are discussed here as group process issues and screen issues. Kozlowski and Holmes (2014, 2017) introduced the concept of role confusion which encapsulated that online group therapists tended to adopt the role of teacher and online group members adopted the role of student. Kozlowski and Holmes (2017) advised that the encouragement of cross-talk style communications between members help to overcome role confusion. Further, Weinberg (2021) discussed that the perception of embodiment is not absent in the online space, but different. He added that online group members continue to retain their own bodily sensations during group, but the physical bodies of others are perceived only visually (lacking other bodily sensations such as touch and smell). Kozlowski and Holmes (2014) and Armington et al. (2020) acknowledged that members tend to perceive this negatively, but did recount that reaching out to the screen to communicate tenderness and warmth non-verbally was effective.

Regarding the unique screen issues of online group therapy, Pipkin et al. (2022) and Whittingham and Martin (2020) both encouraged group members to find a place in their home that is not a personal space (i.e., living room or bedroom) as this has the effect of other members feeling uncomfortable and that they are intruding. Further, this space should be quiet, and free from distractions (Kozlowski & Holmes, 2017) and headphones should be worn to minimise members being overheard (Kozlowski & Holmes, 2017). Kozlowski and Holmes (2014) also reported that online group therapists should include the group members background as an extension of their identity (as it is likely the member has been deliberate as to what items from their personal space are in view) and explore the meaning of this if and when appropriate. A common theme was the added distractibility of seeing one’s self on screen (Kozlowski & Holmes, 2014, 2017; Pipkin et al., 2022; Shaw, 2020; Vaimberg & Vaimberg, 2019; Weinberg, 2021) which can be addressed by turning off the view of self when using Zoom. Finally, the increased self-care needs by both the online group therapist and group members (Amulya & Manickam, 2020; Armington et al., 2020) was identified.

Overall, these findings confirm and extend on Weinberg’s (2021) summary of skills and techniques for effective online group therapy. The findings also reinforce declarations made by Kozlowski and Holmes (2014), Weinberg (2019a, 2019c) and Trachtenberg et al. (2020) that online group therapy and in-person group therapy are different therapy modalities. It is argued that both in-person group therapy and online group therapy are best understood and practiced with some shared theories and competencies, but also with unique theories and competencies to each modality of practice.

What is needed going forward is research identifying the viability of online group therapy by members and therapists when there is shared consensus that online group therapy is a unique therapy modality. Further, research is needed on the effectiveness of online group therapy when facilitated by an online group therapist who has received training on facilitating online group therapy which incorporates the skills and techniques summarised in this review. Research is also needed to initially replicate the suggested techniques above to increase group cohesion and therapeutic presence online, with ongoing research on skills and techniques to improve these processes. Finally, there is little known about the member dynamics in the online group space, and how to manage these dynamics, which future research may seek to investigate.

This review is not without its limitations, including the inclusion of findings from papers appraised as weak in credibility and findings only capturing literature published in English over the last decade. However, there are important implications and recommendations provided for conducting online group therapy. It is recommended that group practitioners, who pivoted their group therapy online during the pandemic, be given an avenue to share their experiences that may confirm findings from this review or add to these findings.

In conclusion, the systematic literature review has been successful in collating all that is known within the published and grey literature databases regarding the practice of delivering group therapy in the online space. This is believed to be the start of a comprehensive body of knowledge informing group practitioners in online group therapy, professional accreditation bodies for developing competencies in this area, and counselling educators for teaching online group competencies in the curricula. The implications of this research include ensuring that the skillset of group therapists is sustainable, and is ethically transferable to the online space for the delivery of essential mental health services, despite inevitable disruptive global events in the future.

## Appendix A

See Tables 2 and 3.

**Table 2:** Paper and quality appraisal details

Author	Paper type	Group details	Findings	QA tool and rating
Adamlje and Jenko (2020)	Expert Opinion	Psychiatric outpatient group therapy (N=8), weekly, for 4 months, using Zoom software.	Absences were equal or lower than in-person groups. Online groups always started 5 minutes early to deal with any technical difficulties, which always occurred. Essential needs include a good internet connection, safe and private space, headphones, microphone, and good quality device. Group members soon compensated for loss of non-verbal communication. Member appearance was more relaxed when online, and background distractions were common. The author stresses the importance of maintaining group rules to minimize these extra transgressions. Group members were more easily immersed in the group experience, benefiting the group growth. Silences are more common, especially toward the latter half of the group program. Group members often commented that “something was missing” online, that is not missing when in-person (e.g., physical proximity). Group process was more important than the content of the group.	JBI-TO +++
Amulya (2020)	Book Chapter	Self-care counselling group (N=8), bi-weekly for 4 weeks, 2 hours duration, using Zoom software.	Online groups overcome barriers such as location and timing. A sense of universality is increased. Other benefits are anonymity and affordability. Interpersonal connectivity was possible, even online. Technical glitches were discussed for their meaning. Increased distractions and interruptions occurred in the online context, and many breaks were included to minimize “zoom fatigue”.	JBI-TO +
Armington et al. (2020)	Expert Opinion	Therapy group, 2 sessions only (N = 75), using Zoom software.	Connection can be improved with reaching gestures towards each other on screen; as well as naming the anxiety related to working online. Finally, online made implicit past roles less likely to re-emerge, making working in the here-and-now easier.	AACODS +
Arrow et al. (2021)	Original Research	CBT Therapy Group (N = 24), 2-hour duration, weekly, for nine weeks, using Blackboard Collaborate software.	Group cohesion (engagement) increases linearly over time and conflict fluctuates over time, mirroring trends in in-person groups.	JBI-QE +
Balem (2021)	Expert Opinion	Psychodrama groups (N = 4), 2-hour duration, weekly, program duration unstated, using zoom software	Importance of confidentiality, use of headphones, and group member responsibility for internet connection. Reported on zoom fatigue.	JBI-Qual +
Ball (2020)	Newspaper Article	Open group for men. Weekly, ongoing, using Google Hangouts.	No details	Exclude
Brown et al. (2020)	Original Research	Task group (N = 3–6), weekly, for 8–14 weeks. Unknown software	Reliance on technology was positively related to coordination, member performance, and leadership behaviours.	JBI-QE +
Datlen et al. (2020)	Expert Opinion	Art therapy group (N = 5). Fortnightly, ongoing, using WhatsApp software.	Members more open in their engagement with others, or their method of engagement changed. Members engaged with other members more. Facilitators felt uncomfortable, as though invading clients’ own personal space. One member missed one-to-one interactions that he enjoyed when groups were in-person.	JBI-Qual +
Ellett et al. (2022)	Original Research	Mindfulness group for schizophrenia (N = 17). Weekly, 90 min duration, for 12 weeks, using Zoom software	Members felt increased connectedness to other members, felt the reduced barriers (travel, ability to turn off camera at overwhelming moments, using headphones) were advantages over in-person groups, but included disadvantages such as technical glitches.	MMAT ++
Gullo et al. (2022)	Original Research	Experienced group therapists (N = 307) responded to quantitative survey regarding their experience of facilitating groups online.	Using CFA, a three-factor model emerged including 1) ease of group cohesion, alliance, and self-disclosure; 2) therapists more present, empathic, and focused; and 3) challenges when working through conflict, avoidance, nonverbal communication, seeing own image, and discomfort.	JBI-CS ++
Gurcan et al. (2021)	Expert Opinion	Therapy groups for schizophrenia patients (N = 17), weekly for 6 months, using Zoom software.	Teaching how to use technology prior to and in early stages of group; and attendance rates higher in online groups compared to in-person groups. Communication was linear rather than cross-talking.	JBI-TO ++
Hamadi et al (2022)	Original Research	CBT-E groups for adolescents with eating disorders (N = 8), weekly, 2-hour duration, for 6 sessions, using MS Teams.	Increased anxiety related to seeing selves on screen, but quickly found the cameras an advantage as it made the group more interactive. Members also felt less judged. First 30 minutes should be dedicated to addressing technical issues.	MMAT +++
Holmes et al. (2015)	Original Research	Student counsellors (N = 12) facilitated online and in-person groups, weekly, 1 hour duration, over 12 months, using Google Hangout software.	For all variables, the in-person groups were rated significantly higher than the online groups indicating that the online group facilitators were less comfortable, less safe, less connected, and less present compared to the in-person group facilitators.	JBI-QE ++
Hung et al. (2021)	Expert Opinion	No details	ICTs limited the interaction between children and youth participants due to limited non-verbal communication, and it took longer for interactions to replicate what is observed in in-person groups. Group leaders need to set up expectations about use of cameras early to avoid group cohesion problems, as well as ensuring members join group from a private location. Online groups have increased methods of interaction (whiteboard, chat) which enriches the group. Online groups overcome geographical limitations making this modality a more flexible therapy option.	JBI-TO ++
Joshi et al. (2021)	Expert Opinion	Group therapy sessions for couples in relationship distress (N = 20), each 90 minutes, weekly, for five weeks, using Zoom software.	Facilitating groups using the co-facilitation model (one facilitated the group whilst the other moderated the shared content in chat functions) helped with group processes. Facilitator adaptation to use of camera or other zoom features (e.g., thumbs up) helped with communication and group presence. Members easily discussed sensitive topics via the alternative communication channels (e.g. chat), which increased trust in the group. Technical issues affected the “flow” of sessions. Group cohesiveness increased as the group progressed.	JBI-TO ++
Kozlowski and Holmes (2017)	Expert Opinion	Counselling educators offer opinion of what needs to be included in counselling curricula to prepare students for online group therapy.	Online groups eliminate geographical, gender, and culture boundaries, thereby offering a sense of universality. Need to address linear discussion styles, role confusion, feeling of being superficially engaged, mistrust amongst group members, and feeling disconnected. Many suggestions are offered.	JBI-TO ++
Kozlowski and Holmes (2014)	Original Research	Group therapy for personal growth (N = 6), weekly, for six weeks, using Google Hangout software.	Communication styles were very linear compared to in-person groups. Members were hyperaware of their own background and would use their background to “portray” their individuality, and this added feature was often a distraction. Members spent much time watching themselves on the monitors. Members joined group with a more relaxed presentation compared to in-person groups. The inability to read nonverbal communications created a constant distance and disconnection from each other. Members felt unsafe to share since trust had not developed. There was role confusion, where members adopted “student” roles and leaders adopted “teacher” roles.	JBI-Qual +++
Lovko et al. (2021)	Original Research	Online Group therapy for day hospital patients (N = 28), thrice weekly for one month, using Google Hangout software.	Anything that occurs in a group members background, including a pet appearing on the screen, needs to be discussed and interpreted as it occurs. Therapists feel less competent and less confident when facilitating groups online.	JBI-QE +
Maheu (2020)	Online article	Expert in telehealth blogging her experience of telehealth group therapy	Common confidentiality breaches are summarised that need to be outlined during the informed consent procedures; meeting members prior to group commencing to screen, review group guidelines, teach about the use of technology, and set expectations; disable chat function or use only for technical difficulties; disable recording of sessions; ensure members have adequate lighting; be consistent with location when online; use headsets, etc.	AACODS +
Marmarosh et al. (2020)	Expert Opinion	Group therapy experts review current evidence of the efficacy of online group therapy	Group members generally report a high degree of satisfaction, and it is important during times of crisis (such as a pandemic). Online groups are less stigmatizing, time efficient, but have challenges to confidentiality, connectedness, and nonverbal communication. They also introduce new challenges, such as the meaning of a member’s background. Online group leaders can model interpersonal feedback, tolerance to conflict, embracing vulnerability, etc, to improve its effectiveness.	JBI-TO ++
Parks (2020)	Expert Opinion	Literature review of relevant articles within a special issue of APA high ranking journal (Group Dynamics: Theory, Research and Practice).	With lack of body language, members tend to focus on communication content and tone to infer others’ feelings and reactions, however the risk of misinterpretations is always elevated when online. This can be mitigated by teaching members about the impact of emotions in group, challenges to accurately detecting emotions in online groups, and strategies for regulating the emotional climate of the group. Also important for members to have a common understanding of the technology, and a belief that it is adequate for the task.	JBI-TO ++
Pipkin et al. (2022)	Original Research	Compassion-focused online group therapy for gender non-conforming persons (N = 8), weekly 2-hour sessions for 8 weeks, using MS Teams.	Facilitator’s use of self-disclosure, modelling, and experiential emotion-focused exercises contributed to a “depth” in group. Group members had anxiety related to the online group format, including problems with technology, talking over others, and seeing self on screen. Group members felt “intrusive” and “uncomfortable” if other members joined from their bedrooms.	JBI-Qual +++
Shaw (2020)	Expert Opinion	Clinician’s reflections of doing art therapy online for adolescents diagnosed with Anorexia Nervosa (N = 3). Groups were weekly for 1-hour, over 7 weeks, using MS Teams.	Protocols were set up early in case a group member became distressed (digitally sent out of room for a few minutes and called by phone). Group members were uncomfortable online due to being able to see self on screen. Attunement to members was hindered due to lack of eye contact. Members felt increased safety since they could “pretend” other group members were not there. Cofacilitators could not communicate with each other nonverbally, so all their communications were verbal and thus transparent to the group.	JBI-TO ++
Trachtenberg et al. (2020)	Original Research	Cognitive-expressive online group for cancer survivors (N = 6-8). Groups were weekly for 90 min, for 8 weeks, using a bespoke online platform (Cancer Chat Canada).	Even though body language is absent, the online group member is not disembodied. Instead, group members have an “embodied presence” in that the experience of their own body is present, but the experience of other body’s is absent. To promote universality, facilitators would share group reflections in the chat function. To promote cohesion, again facilitators would summarise emerging themes in the chat function, encouraged non-linear texting interactions that replicate natural discussions, and typed brief visualizations that focused on bringing members together in a shared space.	JBI-Qual +
Vaimberg et al. (2019)	Book Chapter	No details	Online groups are unique in that the members can see not only the group members, but also themselves, which influences the characteristics of the online group communication.	JBI-TO ++
Van Dijk et al. (2020)	Expert Opinion	Schema Therapy online group for older persons diagnosed with comorbid affective disorders and personality problems (N = 4). Groups were twice weekly, over 20 weeks, using Webex technology.	Members were contacted prior to group to teach the technology, ensure patients understood rules, knew how to ensure their privacy, and used an appropriate space (not bedroom or living room). Members attended 100% of sessions and considered sessions very serious. Soon, patients missed the “informal contact” between sessions, so “break out time” was added.	JBI-TO ++
Weinberg (2019)	Book Chapter	No details	Recommended to do online pre-group meetings with each group members to explain technical issues and online etiquette. Recommends members do not have out of the group relationships, even online. The facilitators technical expertise becomes the holding environment for group members, so ensure you know how to solve simple technical problems. Do not ignore on-screen transgressions, even in the background, instead including them in process discussions. Technical issues and communication failures are part of the dynamics, so include them in discussions. When using Zoom, gallery view is preferred, and select “hide-self-view”. Discourage the use of the chat function, joining via a smart phone, or joining whilst in a car. Facilitators can increase presence by appropriate self-disclosure (related to the here-and-now), acknowledging mistakes, and identifying possible member feelings via non-verbal communication. Avoid “star-shaped” communication styles and encourage cross talk.	JBI-TO ++
Weinberg and Rolnick (2019)	Book Chapter	No details	In online groups, attention to process is critically important. Thus, don’t ignore things that happen (such as technical glitches or failures, background indiscretions). This helps the group develop deeper and be successful.	JBI-TO +
Weinberg (2019a)	Book Chapter	No details	Common difficulties to navigate include group members not being able to identify who each member is looking at, and members cannot locate the gaze of the leader. Another problem is when all members talk at the same time.	JBI-TO ++
Weinberg (2019b)	Book Chapter	No details	The intimacy between members created online has congruence with the intimacy created between members in large groups. Group sense of belonging and cohesion is possible, but just takes longer. Universality is strengthened online due to barriers created by geography, diversity, and cultures being less prominent. The facilitators ability to hold the group comes down to their technical expertise. Group leaders should be aware of the deliberate background choreographed by members and explore its meaning when appropriate. When a boundary crossing has been noticed (even in the background), the facilitator can recruit the help of the group to respond. Always have preparation meetings prior to the group program commencing to discuss rules and educate about the technology. Facilitators can make up for the inability to use the gaze to draw out members by using appropriate self-disclosure (here-and-now). Connectedness is increased with the facilitator’s acknowledgement of mistakes in online groups, particularly when trying to identify affect from non-verbal communications.	JBI-TO +
Weinberg (2020)	Expert Opinion	No details	The online therapeutic alliance has been reported as either inferior or equivalent to that in in-person groups. Online groups not appropriate for those in crisis or those who are easily dysregulated. Online groups are preferred over in-person groups for those with intimacy problems, those with dismissive-avoidant attachment styles, dissociative symptoms, social anxiety, and borderline personality disorder. Online groups are less stigmatizing, overcome barriers to attendance, but do require a computer and access to internet. Pre-group meetings are necessary to ensure members join from a private space. The disadvantage of working in a disembodied environment (no eye contact, no pheromones) can be replaced with verbal transparent communications between co-leaders in the presence of members. Online, facial expressions are enhanced, and group leaders can train themselves to read facial expressions more accurately. Group leaders can also ask members to report on their body sensations, or create physical distance between them and screen, to communicate affect. Leaders presence can be enhanced via increased self-disclosure (here-and-now), taking responsibility for mistakes and empathic failures, and use of imagination.	JBI-TO ++
Weinberg (2021)	Expert Opinion	No details	Group cohesion is possible online, but the development is slower due to technical glitches, linear communication styles, confidentiality problems, dissociative defenses, and no small talk between members outside of group. Members need to be instructed on how to gain confidentiality when joining group from their own settings in pre-group preparation meetings. Technical glitches and failings carry psychological meaning and thus must be discussed. The environment differs due to the “embodied presence” (aware of own body but lack the awareness of others’ bodies except for facial expression). Online group leaders can relate more to facial expressions, and query meaning behind them (not to be overused). The presence of own face on screen can be disrupting. Leader presence can be enhanced with self-disclosure, acknowledging mistakes and empathic failures, and using imagination. Pay attention to the orchestrated background of members, as well as distractions that occur in a member’s background. Pay attention to relaxed attendance (joining when in bedroom, in pajamas, unshaven, unwashed, etc).	JBI-TO ++
Whittingham and Martin (2020)	Online article	No details	Summarises the common breaches to confidentiality in online groups and stresses the importance of alerting members to these early in group. Encourages the importance of using a collaborative platform that is compliant. Emphasises the need to have pre-group screening appointments, and the common group rules for online therapy groups.	AACODS ++

*QA* Quality Appraisal, *JBI-QE* JBI Checklist of Quasi-Experimental Studies (Non-Randomised Experimental studies), *JBI-CS* JBI Checklist for Analytical Cross-Sectional Studies, *JBI-Qual* JBI Checklist for Qualitative Research, *JBI-TO* JBI Checklist for Text and Opinion, *MMAT* Mixed Methods Appraisal Tool, *AACODS* The AACODS Checklist

**Table 3:** Themes and sub-themes: a summary

**Theme**	**Sub-themes**	**Main findings**
Online Vs F2F	Group member viability	Acceptance
		Advantages
	Group leader viability	Acceptance
		Advantages
Group planning	Scheduling	Frequency
		Duration
	Screening	More details
		Group size
	Pre-group preparation	Necessary
	Online group leadership	Co-facilitation
		Communication lines
	Online group rules	Emphasised more
		Unique online rules
Group processes	Therapeutic presence	Diluted
		Self-disclosure, transparency
	Group cohesion	Longer to establish
		Easier to lose
	Holding environment	Privacy
		Technical expertise
		Don’t ignore small incidents
	Member dynamics	Silences
		Technology as escape
		Anger
		Depth
		Inauthenticity
Micro-skills	Verbal communication	Linear
	Non-verbal communication	Difficult
		Members soon adjust
		Gestures or voice
Ethics	Safety	Member's feel less safe
		Members adapt to increase safety
	Boundary breakdowns	Increased casualness
		Increased distractedness
	Confidentiality	More difficult
		Added breaches
Technology	Software	Zoom, Teams, WhatsApp, Webex
	Hardware	Internet
		Devices
		Not mobile phone
	Issues	Time consuming
		Creates distraction and discomfort
		Need to be addressed
	Chat function	Limit or disable
Unique to online	Role confusion	Student/teacher
		Encourage cross talk
	Embodied presence	Non-body presence
		Difficult to create warmth
		Reaching out with hands
	Members physical space	Private and dedicated
		Headphones
		Background
		Camera position
	Self/others on screen	Distracting
		Dysphoric
		Turn off self view
		Facial expressions improved
	Self-care	Zoom fatigue
		After group
